# No causal link between changes in hand position sense and feeling of limb ownership in the rubber hand illusion

**DOI:** 10.3758/s13414-015-1016-0

**Published:** 2015-11-10

**Authors:** Zakaryah Abdulkarim, H. Henrik Ehrsson

**Affiliations:** Brain, Body and Self Laboratory, Department of Neuroscience, Karolinska Institutet, Retzius väg 8, 17177 Stockholm, Sweden

**Keywords:** Modularity of perception, Multisensory processing, Spatial localization

## Abstract

The rubber hand illusion is a perceptual illusion in which participants experience an inanimate rubber hand as belonging to their own body. The illusion is elicited by synchronously stroking the rubber hand and the participant’s real hand, which is hidden from sight. The feeling of owning the rubber hand is accompanied by changes in hand position sense (proprioception), so that when participants are asked to indicate the location of their (unseen) hand, they indicate that it is located closer to the rubber hand. This “proprioceptive drift” is the most widely used objective measure of the rubber hand illusion, and from a theoretical perspective, it suggests a close link between proprioception and the feeling of body ownership. However, the critical question of whether a causal relationship exists between changes in hand position sense and changes in limb ownership is unknown. Here we addressed this question by devising a novel setup that allowed us to mechanically manipulate the position of the participant’s hand without the participant noticing, while the rubber hand illusion was being elicited. Our results showed that changing the sensed position closer to or farther away from the rubber hand did not change the strength of the rubber hand illusion. Thus, the illusion is not dependent on changes in hand position sense. This finding supports models of body ownership and central body representation that hold that proprioceptive drift and the subjective illusion are related to different central processes.

The rubber hand illusion is a perceptual illusion in which participants experience an inanimate rubber hand as being their own hand (Botvinick & Cohen, 1998). The illusion is elicited by synchronously stroking the rubber hand, which is placed in an anatomically plausible location, and the participant’s real hand, which is hidden from sight. After a stimulation period of approximately 10–15 s, most participants begin to sense touch at the location where they see the rubber hand being stroked and develop the feeling that the rubber hand is their own hand (Ehrsson, Spence, & Passingham, 2004; Lloyd, 2007). Over the last decade, the rubber hand illusion has become a popular model system for investigating central body representation, the sense of bodily self, and the integration of visual and somatosensory information from the body. However, the causal processes that underpin the illusion have remained unclear, and the best means of objectively measuring the illusion have been intensely debated (de Vignemont, 2011; Ehrsson, 2012; Makin, Holmes, & Ehrsson, 2008; Moseley et al., 2008; Rohde, Di Luca, & Ernst, 2011; Rohde, Wold, Karnath, & Ernst, 2013; Tsakiris, 2010). In their original report, Botvinick and Cohen employed two measures of the illusion. A subjective measure was obtained by having participants rate a set of statements that were devised to capture various aspects of the perceptual illusion on a visual analogue scale, such as “It felt as if the rubber hand was my hand” and “It seemed as if I were feeling the touch of the paintbrush in the location where I saw the rubber hand touched.” A more objective measure was constructed by having participants close their eyes after the induction of the illusion and point toward their hand, the so-called “proprioceptive drift test.” Botvinick and Cohen found a pointing bias toward the rubber hand after as compared with before the induction of the illusion (“proprioceptive *drift*”), and that this bias was greater when the hands were stroked synchronously as opposed to asynchronously (“proprioceptive *shift*”; i.e., the difference in proprioceptive drift between the two conditions). The discovery of proprioceptive drift was regarded as evidence for a model of the trimodal integration of vision, touch, and proprioception (Botvinick & Cohen, 1998). According to this model, changes in proprioception are an integral part of the illusion phenomenon (Ehrsson, 2012). Following the original report, the proprioceptive drift test quickly became the most commonly used objective test of the illusion (Tsakiris, 2010). This popularity stems not only from the fact that the test is easy to administer and that the degrees of proprioceptive drift (Botvinick & Cohen, 1998; Costantini & Haggard, 2007; Ehrsson, Holmes, & Passingham, 2005; Kalckert & Ehrsson, 2014; Longo, Schüür, Kammers, Tsakiris, & Haggard, 2008; Samad, Chung, & Shams, 2015; Tsakiris & Haggard, 2005; Tsakiris, Prabhu, & Haggard, 2006) and shift (Guterstam, Gentile, & Ehrsson, 2013) across participants have been reported to correlate with the subjective ratings of limb ownership, but also from the commonly held assumption that the drift in hand position sense toward the model hand reflects a causal factor in the generation of the rubber hand illusion.

However, the validity of proprioceptive drift as a behavioral proxy for measuring the illusion has been questioned. This concern stems in part from the fact that changes in hand proprioception can occur without changes in limb ownership (Holmes, Crozier, & Spence, 2004; Holmes, Snijders, & Spence, 2006; Makin et al., 2008), and in part from the fact that some rubber hand illusion experiments have failed to detect correlations between proprioceptive drift and subjective ratings of the illusion (Holle, McLatchie, Maurer, & Ward, 2011; Rohde et al., 2011). Of particular relevance to this concern is a study by Rohde and colleagues, who presented data suggesting that synchronous visuotactile stimulation does not strengthen proprioceptive drift but, rather, that asynchronous visuotactile stimulation in the classical control condition eliminates it. These authors proposed that proprioceptive drift occurs as a default when a body part is placed in view, irrespective of whether limb ownership is felt, and that asynchronous visuotactile stroking offers contradicting information that halts this independent process of visuo-proprioceptive recalibration (Rohde et al., 2011). However, these authors made no attempt to falsify the proposition that an ownership illusion requires a change in hand position sense toward the rubber hand, nor did they investigate the possible causal relationship between changes in hand position sense and the rubber hand illusion.

What is lacking in the literature thus far is a study that directly examines the possible causal role of proprioception in the rubber hand illusion by experimentally manipulating sensed hand position during the period when the illusion is being elicited. Would an externally induced change in hand position sense toward the rubber hand increase the strength of the illusion? Conversely, would changing the sense of hand position away from the rubber hand reduce the illusion? If hand position sense constitutes a causal factor in the illusion, one would expect a positive answer to these two questions. By contrast, a negative answer to both would indicate that proprioceptive drift occurs as a consequence of the subjective illusion or that it constitutes an altogether independent process (i.e., effectively an epiphenomenon).

In the present study, we addressed this question by developing a novel setup that allowed us to physically manipulate the location of the participant’s real hidden hand, without the participant noticing this manipulation, as we induced the rubber hand illusion, thereby effectively simulating proprioceptive drift toward or away from the model hand. To achieve this, the participant’s hand was placed on a small table that was very slowly and gently pulled by a motor toward or away from the rubber hand while the rubber hand illusion was being induced by synchronized brush stroking (or with asynchronous stroking, in the control condition). In the first experiment, we examined whether changing the sensed hand position toward or away from the rubber hand increased or decreased the illusion, respectively. In the second experiment, we compared the effect of changing sensed hand position away from the rubber hand to the effect in the classical condition with the participant’s hand completely immobile. In both experiments, questionnaires were used to quantify the subjective illusion, and the proprioceptive drift test was administered in line with earlier studies. Collectively, our results suggest that experimentally manipulating hand position sense has no effect on the rubber hand illusion. This result opposes the hypothesis that the illusion is caused by changes in proprioception. Our finding has a bearing on existing and emerging neurocognitive models of body ownership and central body representation (Armel & Ramachandran, 2003; Blanke, 2012; Botvinick & Cohen, 1998; Ehrsson, 2012; Makin et al., 2008; Moseley, Gallace, & Spence, 2012; Samad et al., 2015; Tsakiris, 2010).

## Materials and method

### Ethics statement

All participants provided their written informed consent prior to their participation. All experiments were approved by the Regional Ethical Review Board of Stockholm.

### Participants

A total of 36 naïve, healthy adult participants were recruited for the two experiments (18 for each experiment), with the following age and gender distributions: Experiment 1, 18 participants (nine females, nine males; mean age 29 ± 11 years); Experiment 2, 18 participants (eight females, ten males; mean age 28 ± 15 years). All participants were recruited from the student population in Stockholm. The participants received a cinema ticket as compensation for their participation in the experiment.

### Setup, mechanical hand displacement apparatus, and visuotactile stimulation

#### Mechanical hand displacement apparatus

**Fig. 1:**
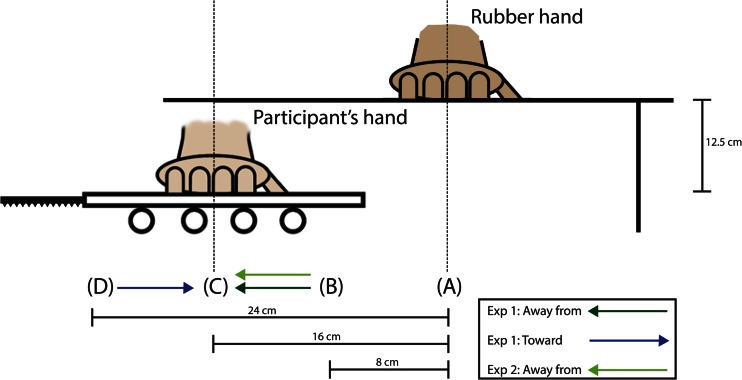
Schematic illustration of the setup employed in both experiments. The rubber hand was placed on top of a small table, and the participant’s hand was placed 12.5 cm underneath the rubber hand on the mechanical hand displacement apparatus. Point A indicates the position of the rubber hand, B and D indicate the different start positions of the participant’s hand, and C indicates the end position. The arrows indicate the positions between which the participant’s hand was displaced in the various conditions of the two experiments. In the static condition of Experiment 2, the participant’s hand remained static at Position B

An apparatus designed to displace the participant’s right hand was devised. The apparatus consisted of two sheets of Plexiglas glued together and separated by Styrofoam. The lower sheet rested on a set of plastic cylinders that in turn rested on a rubber mouse pad (Fig. 1). The lower Plexiglas sheet was connected to an electrical engine (Micro Motors E192.24.625, Verderio Inferiore, Italy) via a cogwheel and a rack bar. This allowed the engine to displace the Plexiglas sheets both laterally and medially at a velocity of 0.9 mm per second, causing an 8-cm displacement to take approximately 90 s. On the basis of previous studies on the sensitivity of limb kinesthesia, it has been reported that adults do not reliably sense passive angular joint displacements that are slower than 0.3 deg per second (Pickett & Konczak, 2009). The speed of the extension or flexion in the elbow joint in this experiment was aimed at being less than or equal to 0.3 deg per second.

#### Basic rubber hand illusion setup

The experiment was conducted in a soundproof testing room (40-dB noise reduction). The participants sat on a comfortable chair throughout the entire experiment. The rubber hand illusion was induced by synchronously stroking the participant’s right hand, which was placed underneath a small table, and a cosmetic prosthetic right hand, which was placed on top of the small table. The participant’s right hand thus rested on the upper Plexiglas sheet of the mechanical hand displacement apparatus (which was placed underneath the small table) 12.5 cm below the surface on which the rubber hand rested. Furthermore, the real hand was always placed lateral to the rubber hand (never vertically aligned), and the starting position of the participant’s right hand on the horizontal plane differed across trials (see the specific Method section below for each experiment). A black cloth was taped to the table to cover the stump of the rubber hand and was tied around the participant in a manner resembling a barber’s gown, to give the impression that the rubber hand was continuous with the body.

The visuotactile stimulation consisted of strokes with two small paintbrushes (1 cm wide). The brushstrokes were delivered to all fingers and to the back of the hand with a frequency of one brushstroke per second. The average length of each brushstroke was approximately 8 cm (from the metacarpophalangeal joint to the fingertip), and the brushstrokes were delivered randomly on all digits. There were two different modes of stimulation, synchronous and asynchronous. In the *synchronous* condition, the experimenter brushed the rubber hand and the participant’s real hand simultaneously. In the *asynchronous* condition, the rubber hand was touched first and the participant’s real hand was touched following a delay of approximately 500 ms. The asynchronous condition thus served as a control condition for the illusion.

### Outcome measures

#### Experimental questionnaire

The questionnaire consisted of nine statements regarding the participant’s experience, on a 7-point Likert scale ranging from −3 to 3, with −3 corresponding to *fully disagree* and +3 corresponding to *fully agree*. The statements were adopted from Botvinick and Cohen’s (1998) original report and were modified slightly to fit the purposes of this study (Table 1). Among the nine questionnaire items, items S1–S3 were aimed at capturing the specific illusory experience, and items S4–S9 served as controls for the perception of movement, task compliance, and suggestibility. To quantify the strength of the illusion, we computed an “illusion index,” which was defined as the difference between the means of the pooled illusory statements (S1–S3) and the control statements (S4–S9).

**Table 1:** Statements used in the questionnaire for both experiments

S1	It seemed as if I were feeling the touch of the paintbrush in the location where I saw the rubber hand touched.
S2	It seemed as though the touch I felt was caused by the paintbrush touching the rubber hand.
S3	I felt as if the rubber hand were my hand.
S4	It felt as if my real hand were drifting toward or away from the rubber hand.
S5	It seemed as if I might have more than one right hand or arm.
S6	It seemed as if the touch I was feeling came from somewhere between my own hand and the rubber hand.
S7	It felt as if my real hand were turning “rubbery.”
S8	It appeared as if the rubber hand were drifting toward or away from my real hand.
S9	The rubber hand began to resemble my own real hand, in terms of shape, skin tone, freckles, or some other visual feature.

S1–S3 aimed to capture specific aspects of the rubber hand illusion. S4 was a control statement that aimed to capture whether the participants had consciously perceived any displacement, illusory or veridical. S5–S9 served as control statements for suggestibility and task compliance

#### Proprioceptive drift and proprioceptive shift

Proprioceptive drift was measured by having the participants close their eyes and raise their left hand, while their right hand was placed underneath the small table. The experimenter then placed the participant’s left index finger on the distal (from the participant’s point of view) horizontal edge of the small table, and the participant had to slide the left index finger along the edge until he or she felt that the finger was precisely above the tip of the right index finger, at which point the participant would stop and utter the Swedish word for “here.” The starting point on the distal edge of the small table from where the participants were to make the finger location judgment was varied randomly, to prevent participants from memorizing the movement itself. Proprioceptive drift was calculated by subtracting the reported position of the right index finger before the induction of the rubber hand illusion from the reported location of the right index finger after the induction of the illusion. No corrections were thus made for the veridical hand position at the end of each trial when calculating the proprioceptive drift. The proprioceptive shift was calculated by subtracting the proprioceptive drift in the asynchronous condition from that in the corresponding synchronous condition, to obtain a synchrony-specific measure of the change in perceived limb location.

#### Postexperiment questionnaire

The postexperiment questionnaire served to probe whether the participants had noticed the displacement of their hand or whether they had discerned the displacement in some other way. The questionnaire consisted of five items (Table 2), and the third item (Q3) was a forced choice question that required participants to answer “yes” or “no” regarding whether they had experienced any movement of their hidden, real hand. If the answer was “yes,” they were asked to specify the movement so that it was possible to draw conclusions about whether the movement experienced was caused by the movement apparatus or merely by confabulation.

**Table 2:** Items on the postexperiment questionnaire

Q1	What do you think the aim of the experiment was?
Q2	In what way do you think the different trials differed?
Q3	Did you at any point during the experiment feel that your real hand was moving? (Y/N) [*forced choice*]
Q4	If “yes” on Q3, please describe the movement.
Q5	Do you have other comments regarding the experiment?

The original questionnaire was written in Swedish. Note that Q3 is a forced choice question; that is, the participants had to choose between “yes” and “no.”

### Procedure

Upon their arrival, the participants were seated and given oral information about the experiment, as well as instructions and a demonstration of the proprioceptive drift measurement procedure. The participants were then told to wear a pair of noise-reducing earplugs and on-ear noise-reducing headphones to prevent them from hearing the sound of the electrical engine during the trials. In every trial, the participants were initially instructed to keep their hands resting in their lap. Upon being given the experimenter’s cue, the participants raised their right arm, and the experimenter placed it under the small table on top of the mechanical hand displacement apparatus. The participants then closed their eyes and used their left index finger to slide along the horizontal edge of the small table to the point where they felt that they were exactly at the tip of their right index finger (as part of the proprioceptive drift test described above). When this was completed, the participants rested their left hand on their lap and opened their eyes, and the experimenter began the visuotactile stimulation (see above). The stimulation continued for 90 s while their hidden right hand either was or was not being displaced (depending on the trial and experiment). Subsequently, the participants used their left index finger to indicate the perceived position of their hidden right index finger in a procedure that was identical to the one used before the visuotactile stimulation. The experiment questionnaire was administered after the first unique repetition of each condition, and the postexperiment questionnaire was distributed at the conclusion of the experiment (only in Exp. 2).

### Statistical analysis

The number of participants recruited was based on previous studies on the rubber hand illusion that had used similar outcome measures (Botvinick & Cohen, 1998; Guterstam, Petkova, & Ehrsson, 2011; Rohde et al., 2011; Tsakiris & Haggard, 2005). All statistics were calculated using SPSS for Windows, release 21.0 (IBM Corp., Armonk, NY). The data from every participant were pooled into one dataset per condition. Each dataset was tested for normality using a one-sample Kolmogorov–Smirnoff test. No dataset deviated significantly from normality, and therefore, all analyses were conducted using parametric tests. We used a repeated measures analysis of variance (ANOVA) to test for main effects and interactions, followed by paired *t* tests for specific comparisons. The effect sizes for the ANOVAs were calculated using *η*^2^ = SS_effect_/SS_total_, (SS = Sum of Squares) and the effect sizes for the planned comparisons were calculated using Cohen’s *d*, according to Eq. 8 from Morris and DeShon (2002).

### Experiment 1: Comparing hand displacement toward and away from the rubber hand

The aim of this experiment was to test the causal link between changes in hand proprioception and the feeling of owning the rubber hand. The crucial manipulation in this experiment was that the participant’s real hand was very slowly mechanically displaced toward the rubber hand in half of the trials and very slowly displaced away from the rubber hand in the other half. We hypothesized that displacing the hand toward the rubber hand might strengthen the illusion of ownership because the mechanically induced change in hand position would be in the same direction as the proprioceptive drift related to the illusion, and the two would thus add up. Under the assumption that the change in hand position sense toward the rubber hand plays a causal role in the rubber hand illusion, this experimental manipulation should facilitate the illusion. Conversely, under the same logic, one would expect the subjective illusion to become weaker when the real hand was mechanically displaced away from the rubber hand, because then any illusion-induced proprioceptive drift would be counteracted by a more effective physical displacement of the real hand, so that the hand would be perceived to be increasingly farther away from the rubber hand. The experiment had a within-subjects factorial design.

The participant’s right hand was placed in one of two different starting positions and ended at the same final position, 16 cm lateral to the rubber hand. In the conditions in which the participant’s real hand was displaced toward the rubber hand, the real hand started 24 cm lateral to the rubber hand, and in the conditions in which the participant’s real hand was displaced away from the rubber hand, it started 8 cm lateral to the rubber hand. The rubber hand was placed on top of the table in full view 12.5 cm above the horizontal plane on which the position of the visually occluded real hand was being manipulated. A 2 × 2 factorial design with Synchrony (synchronous vs. asynchronous) and Direction of Displacement (displaced medially vs. displaced laterally) as the main factors was employed in this experiment. Each condition was repeated three times.

### Experiment 2: Comparing hand displacement with a stationary real hand (classical illusion)

The aim of this experiment was to directly compare a condition in which changes in hand position sense were mechanically induced with the classical illusion condition in which the real hand was completely immobile. We chose to compare the classical illusion with mechanical displacement away from the rubber hand because this condition was expected to reduce the illusion, under the hypothesis that proprioception plays a causal role in the illusory experience. We reasoned that this comparison was particularly important, given that the first experiment (described above) had produced a negative result, suggesting that mechanically induced changes in hand position sense toward or away from the model hand had no effect on the illusion experience (see the Results section for details). By comparing a hand movement condition with the classical condition with a static hand, we could rule out the possible general suppression of the illusion by hand movement, regardless of direction.

In this experiment, the participant’s right hand was placed at the same starting position 8 cm lateral to the rubber hand for all trials. In the trials in which the hand was displaced away from the rubber hand, it was displaced 8 cm laterally to a final position 16 cm lateral to the rubber hand. In the static condition, the real hand was placed 8 cm lateral to the rubber hand and was not moved, remaining completely immobile throughout the entire trial. Again, the rubber hand was placed on top of the table in full view of the participant, 12.5 cm above the plane on which the participant’s real right hand was placed out of sight under the table. A 2 × 2 factorial design with Synchrony (synchronous vs. asynchronous) and Displacement (displaced away from hand vs. static) as the main factors was employed in this experiment. Each condition was repeated three times.

## Results

### Experiment 1: Comparing hand displacement toward and away from the rubber hand

#### Questionnaire ratings

**Fig. 2:**
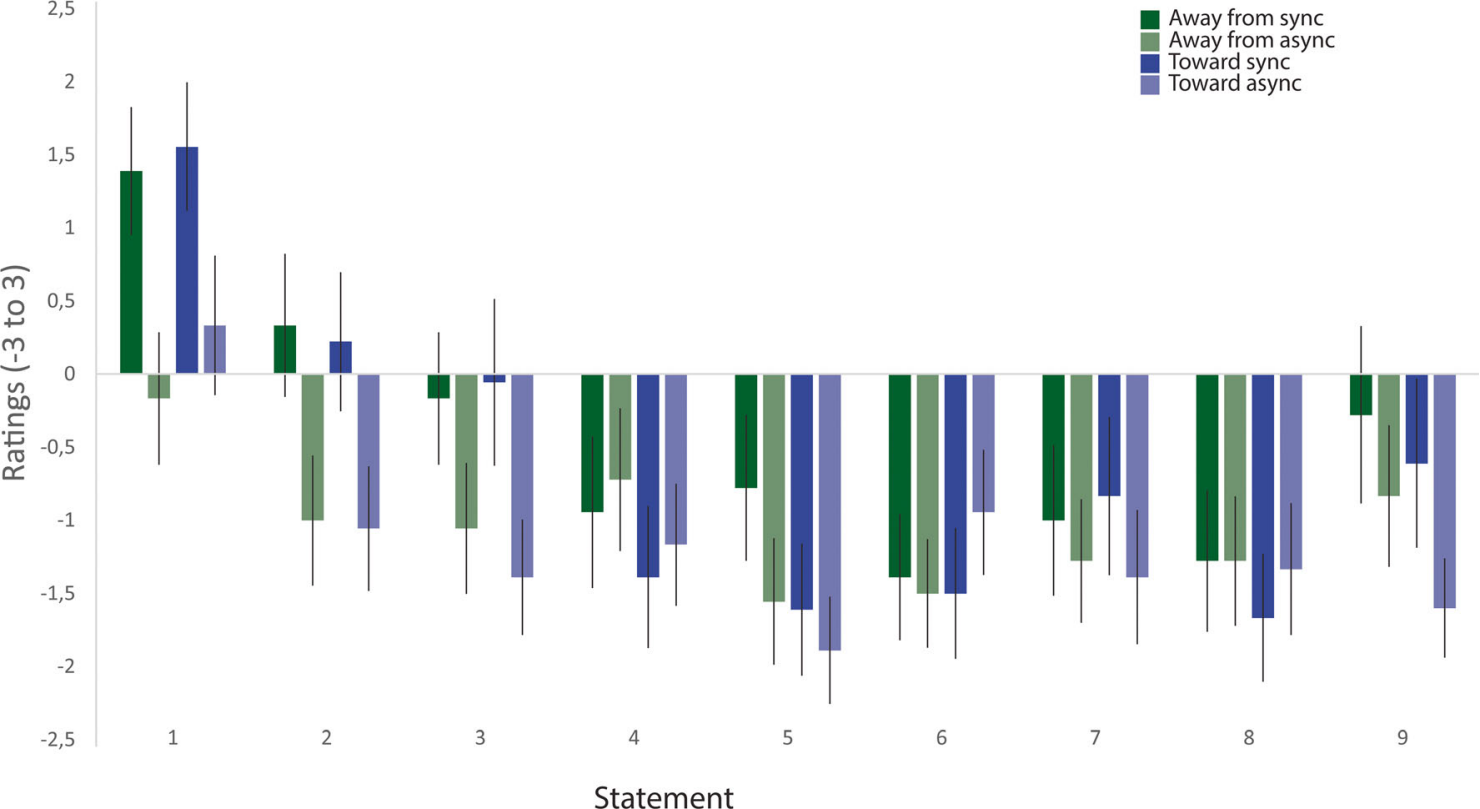
Ratings for the questionnaire statements on a 7-point Likert scale ranging from −3 to +3, with −3 corresponding to *fully disagree* and +3 corresponding to *fully agree*. The bars show the means for the pooled ratings from all participants for each statement and condition. The error bars indicate the standard errors of the means

**Fig. 3:**
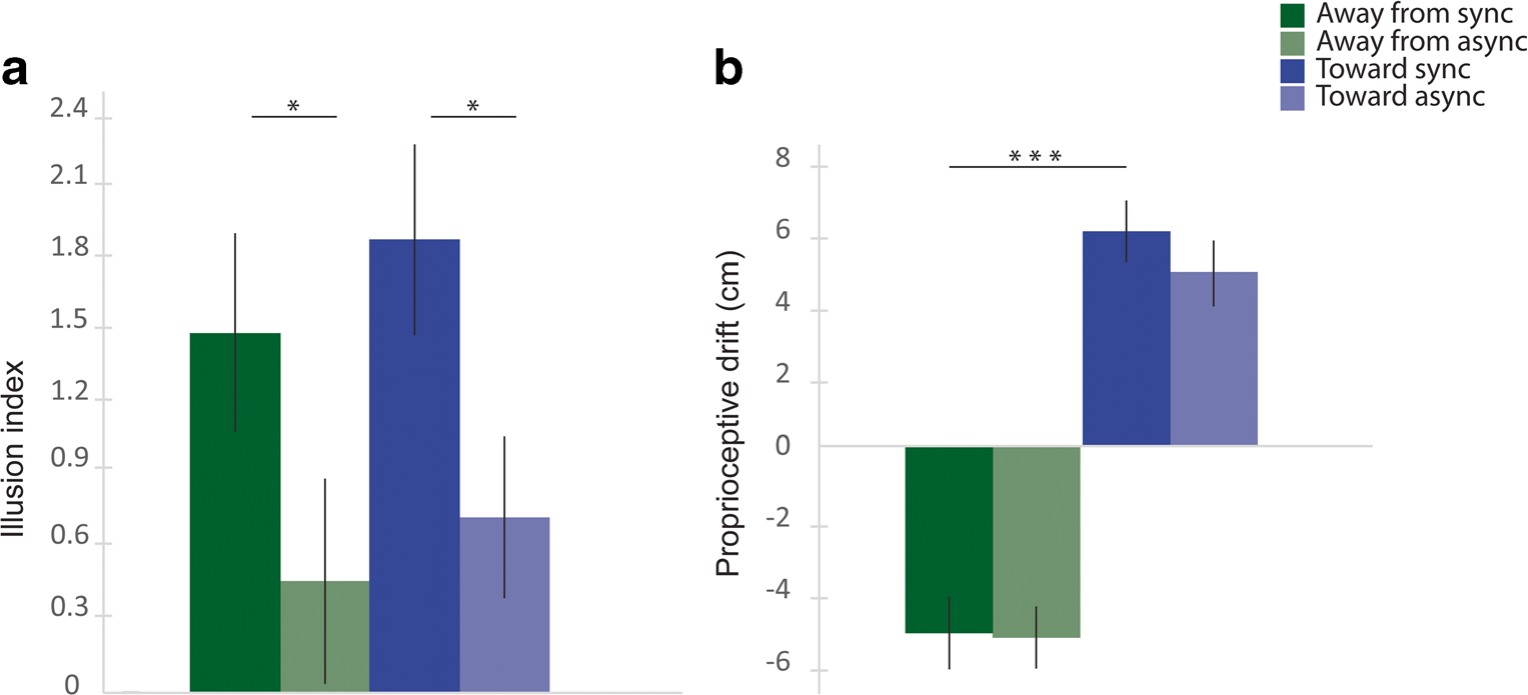
Results from the questionnaire (i.e., illusion index; A) and proprioceptive drift (B) for Experiment 1. The illusion index was calculated by subtracting the means of the ratings for the pooled control statements from the means of the ratings for the pooled illusion statements. Proprioceptive drift was measured in centimeters; values >0 were directed toward the rubber hand, and values <0 were directed away from the rubber hand. Error bars indicate the standard errors of the means. ^*^ *p* < .05, ^***^ *p* < .001

The results from the questionnaire showed higher ratings of the illusion-related statements (S1–S3) in the synchronous than in the asynchronous condition, whereas this difference was not found for the control questions. The questionnaire ratings are depicted in Fig. 2, which shows the means of the pooled ratings from all participants for each statement. The illusion index (also described above), which was defined as the difference between the mean for the pooled illusion statements (S1–S3) and the mean for the pooled control statements (S4–S9), was also analyzed. The illusion indexes were then analyzed with a 2 × 2 ANOVA with the main factors of Synchrony and Direction of Displacement, followed by *t* tests for planned comparisons. The ANOVA revealed a main effect of synchrony [*F*(1, 17) = 12.424, *p* = .003, *η*^2^ = .125], but no main effect of direction of displacement [*F*(1, 17) = 1.321, *p* = .266] and no significant interaction [*F*(1, 17) = 0.042, *p* = .840]. The post-hoc *t* test showed significantly higher illusion indexes for the synchronous than for the asynchronous conditions; for synchronous toward versus asynchronous toward, the values were *t*(17) = 2.641, *p* = .018 (two-tailed), *d* = 0.577, and for synchronous away versus asynchronous away, the values were *t*(17) = 2.449, *p* = .025 (two-tailed), *d* = 0.620. No significant difference in illusion indexes was observed between the two synchronous conditions or between the two asynchronous conditions (Fig. 3A). These results indicate that there were no significant differences in perceived illusion strength between the synchronous conditions.

#### Proprioceptive drift

**Fig. 4:**
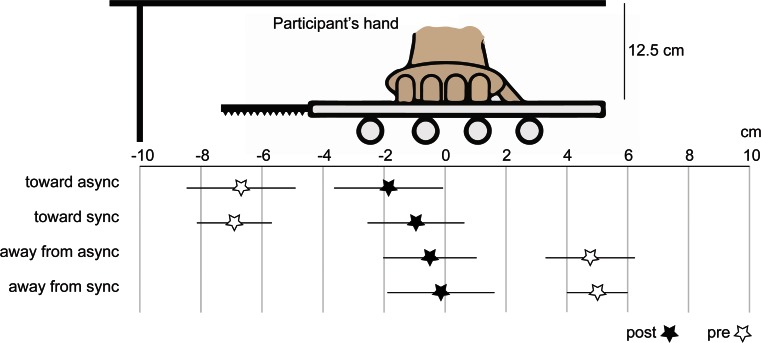
A descriptive illustration of all pointing responses in the proprioceptive drift test in Experiment 1. The indicator scale is in centimeters. Stars indicate the mean estimated right-hand positions, and the lines indicate the standard errors of the means. Both the responses prior to the period of visuotactile stimulation and mechanical hand displacement (“pre”; hollow stars) and the responses after this period (“post”; black stars) are indicated. (The *proprioceptive drift* corresponds to the difference between “post” and “pre,” as we describe in the Method section.) The 8-cm position indicates the starting position for the participant’s hand in the “away” conditions, whereas the −8-cm position indicates the starting position for the hand in the “toward” conditions. The 0-cm position indicates the final position of the hand in all conditions. The rubber hand was placed at 16 cm (not shown in the figure)

The proprioceptive drift task revealed that the right hand was perceived to be located farther away from the rubber hand in the trials in which the participant’s hand was displaced away from the rubber hand and closer toward the rubber hand in the trials in which the participant’s hand was displaced toward the rubber hand (Fig. 3B). The ANOVA yielded a main effect of direction of displacement [*F*(1, 17) = 112.211, *p* < .001, *η*^2^ = .682], but no main effect of synchrony [*F*(1, 17) = 2.102, *p* = .165] and no significant interaction [*F*(1, 17) = 0.771, *p* = .392]. The *t* tests showed a significant difference between the synchronous condition in which the hand was moved away from the rubber hand and the synchronous condition in which the hand was moved toward the rubber hand [*t*(17) = 5.071, *p* < .001 (two-tailed), *d* = 2.612; Fig. 3B; see Fig. 4 for an illustration of the absolute indications of right-hand position]. We observed no significant difference when comparing each synchronous condition with its corresponding asynchronous condition.

#### Correlation between illusion strength and proprioceptive shift

Two correlation analyses were performed on the questionnaire data and the proprioceptive drift data, split up by the Direction of Displacement factor. The analyses were performed by calculating the difference in illusion indexes (see above) between the synchronous and asynchronous conditions and relating this to the difference observed in proprioceptive drift between the synchronous and asynchronous conditions (the proprioceptive shift). The analysis of the “toward” conditions revealed no significant Pearson correlation (*r* = .417, *p* = .085, *df* = 16), and the analysis of the correlation in the “away-from” conditions did not, either (Pearson correlation, * r* = .304, *p* = .220, *df* = 16).

### Experiment 2: Comparing hand displacement with a stationary real hand (classical illusion)

#### Questionnaire ratings

**Fig. 5:**
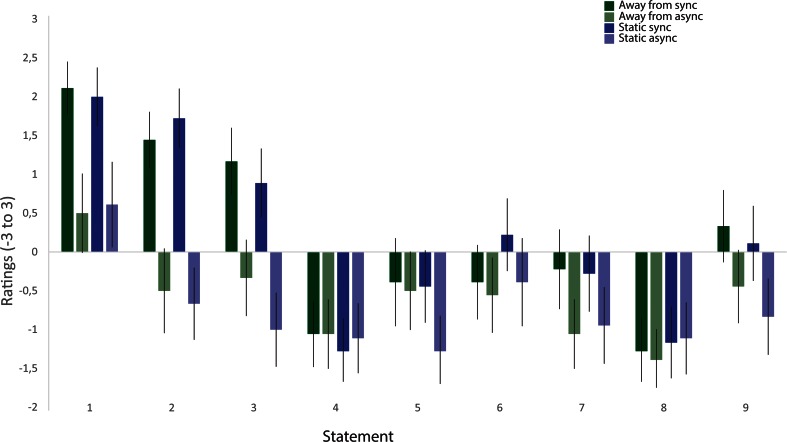
Ratings for the questionnaire statements on a 7-point Likert scale ranging from −3 to +3, with −3 corresponding to *fully disagree* and +3 corresponding to *fully agree*. The bars show the means for the pooled ratings from all participants for each statement and condition. The error bars indicate the standard errors of the means

**Fig. 6:**
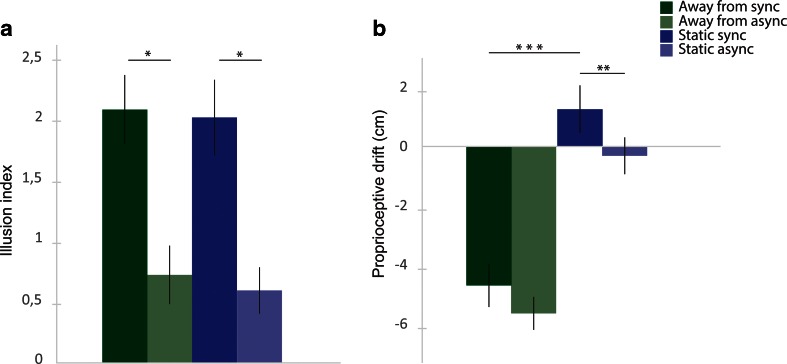
Results from the questionnaire (i.e., illusion index; A) and proprioceptive drift (B) for Experiment 2. The illusion index was calculated by subtracting the means of the ratings for the pooled control statements from the means of the ratings for the pooled illusion statements. Proprioceptive drift was measured in centimeters; values >0 were toward the rubber hand, and values <0 were away from the rubber hand. Error bars indicate the standard errors of the means. ^*^ *p* < .05, ^**^ *p* < .01, ^***^ *p* < .001

The questionnaire results revealed higher ratings for the illusion-related statements in the synchronous than in the asynchronous conditions, whereas this difference was not found for the control questions. The ratings are depicted in Fig. 5, which shows the means of the pooled ratings from all participants for each statement. For the statistical analysis, the illusion index was computed for each condition to quantify the subjective illusion strength, as we described above. A 2 × 2 ANOVA was performed, followed by *t* tests for planned comparisons. The ANOVA revealed a main effect of synchrony [*F*(1, 17) = 25.826, *p* < .001, *η*^2^ = .379] but no significant main effect of displacement [*F*(1, 17) = 0.638, *p* = .443] and no significant interaction [*F*(1, 17) = 0.022, *p* = .884]. Additionally, a significant difference was apparent between the synchronous and asynchronous static conditions [*t*(17) = 4.32, *p* = .001 (two-tailed), *d* = 1.759]. Finally, there was also a significant difference between the synchronous and asynchronous displacement conditions [*t*(17) = 4.065, *p* = .002 (two-tailed), *d* = 1.232]. We found no significant difference between the two synchronous conditions or between the two asynchronous conditions (Fig. 6A). The questionnaire rating results indicate no difference in perceived illusion strength between the conditions in which the participant’s real hand was kept static compared with those in which the hand was slowly mechanically displaced.

#### Proprioceptive drift

**Fig. 7:**
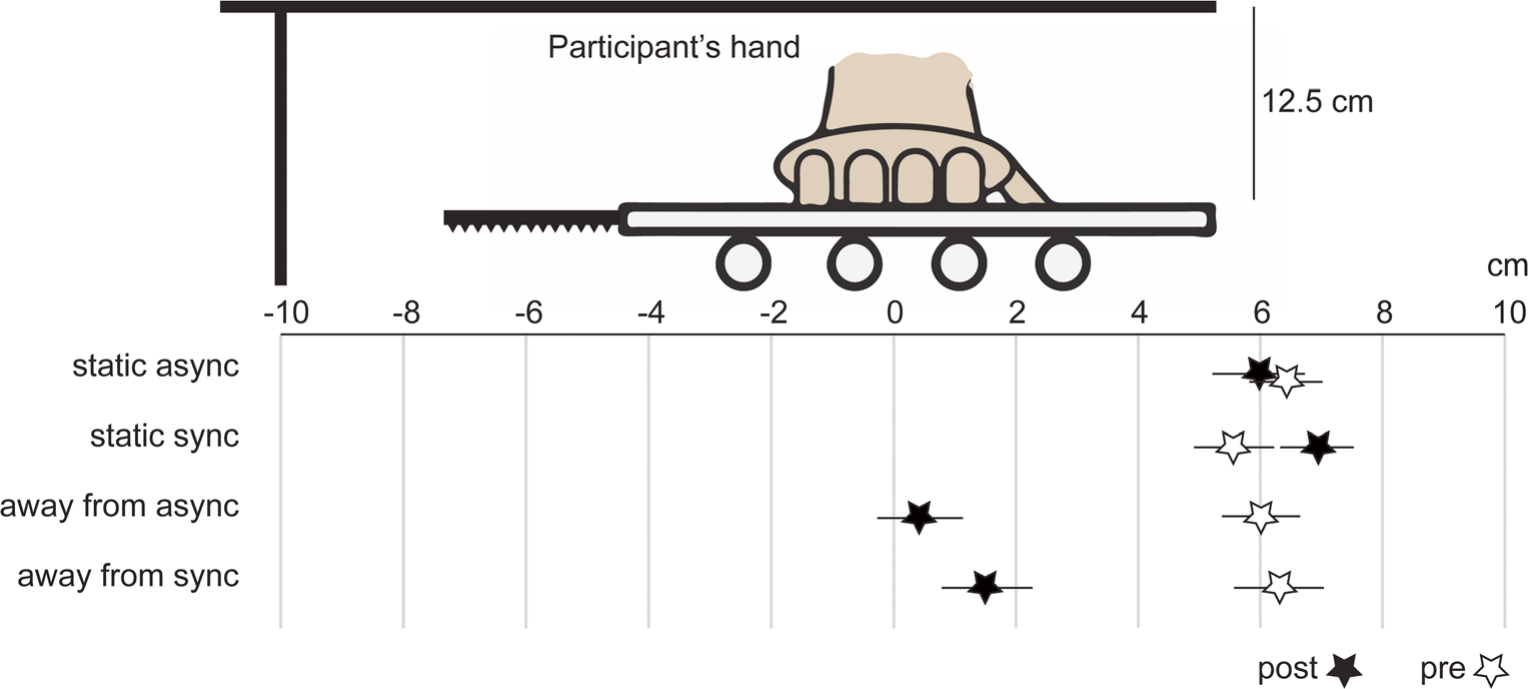
A descriptive illustration of all pointing responses in the proprioceptive drift test in Experiment 2. The indicator scale is in centimeters. Stars indicate the mean estimated right-hand positions, and the lines indicate the standard errors of the means. Both the responses prior to the period of visuotactile stimulation and mechanical hand displacement (“pre”; hollow stars) and the responses after this period (“post”; black stars) are indicated. (The *proprioceptive drift* corresponds to the difference between “post” and “pre,” as we describe in the Method section.) The 8-cm position indicates the starting position for the participant’s hand in all conditions, whereas the 0-cm position indicates the final position in the “away-from” conditions. In the static conditions, the participant’s hand remained at 8 cm throughout the experiment. The rubber hand was placed at 16 cm (not shown)

In this experiment, proprioceptive drift was directed *away* from the rubber hand in the synchronous conditions in which the hand had been mechanically displaced away from the rubber hand. This contrasts to the synchronous static condition, in which we observed a proprioceptive drift *toward* the rubber hand, in line with earlier observations from the classical rubber hand illusion. The proprioceptive drift data were analyzed with a 2 × 2 ANOVA with the main factors Synchrony (synchronous vs. asynchronous) and Displacement (displacement vs. static), followed by paired *t* tests for planned comparisons. The results showed a main effect of displacement [*F*(1, 17) = 251.245, *p* < .001, *η*^2^ = .602] and a main effect of synchrony [*F*(1, 17) = 11.314, *p* = .001, *η*^2^ = .067], but no significant interaction [*F*(1, 17) = 0.982, *p* = .326]. As expected, we obtained significantly higher proprioceptive drift in the synchronous than in the asynchronous static condition [*t*(17) = 1.984, *p* = .003 (two-tailed), *d* = 0.435], in line with earlier studies on the classical rubber hand illusion. We observed no significant difference in proprioceptive drift between the synchronous and asynchronous displacement-away conditions [*t*(1, 17) = 1.943, *p* = .057, two-tailed], although a statistical trend was observed. In addition, we found a significant difference between the synchronous static and synchronous displacement-away conditions [*t*(17) = 12.007, *p* < .001 (two-tailed), *d* = 1.638; Fig. 6B; see Fig. 7 for an illustration of the absolute indications of the right-hand position].

#### Correlation between illusion strength and proprioceptive shift

**Fig. 8:**
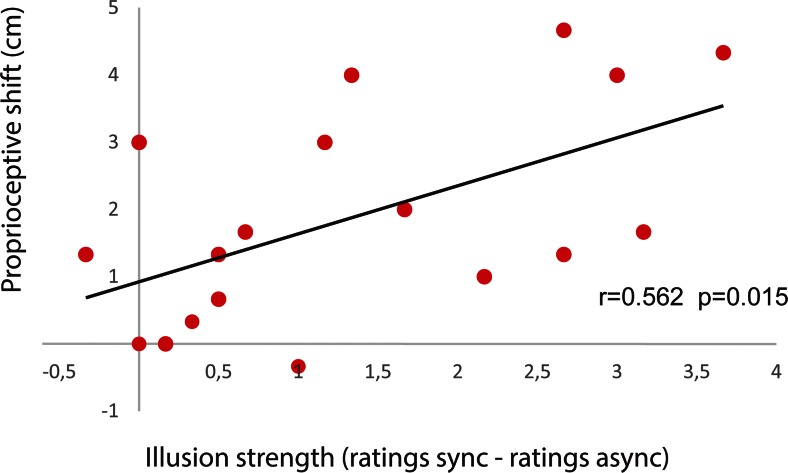
Correlations between illusion strength and proprioceptive shift in Experiment 2. Illusion strength was calculated by subtracting the means of the pooled ratings of the illusion statements in the asynchronous static condition from the means of the pooled ratings of the illusion statements in the synchronous static condition. Proprioceptive shift is indicated in centimeters

Next, a correlation analysis was performed on the proprioceptive drift data and the questionnaire data in the static-hand conditions. The analysis was performed by calculating the differences in the illusion index (see above) between the synchronous and asynchronous static conditions and relating this to the differences observed in proprioceptive drift between the synchronous and asynchronous static conditions (the proprioceptive shift). This analysis revealed a significant Pearson’s correlation [*r* = .562, *p* = .015, two-tailed, *df* = 16], indicating that the greater the increase in subjective illusion in the synchronous than in the asynchronous condition, the greater the proprioceptive drift toward the rubber hand (Fig. 8). We also examined whether a similar relationship could be found between subjective illusion and proprioceptive drift in the conditions in which the real hand was mechanically displaced. However, when we ran the equivalent analysis, we observed no significant correlation between subjective illusion strength and proprioceptive drift in the away-from conditions (Pearson correlation *r* = .123, *p* = .628, *df* = 16; Spearman correlation *r* = .132, *p* = .602, *df* = 16).

#### Postexperiment questionnaire

**Fig. 9:**
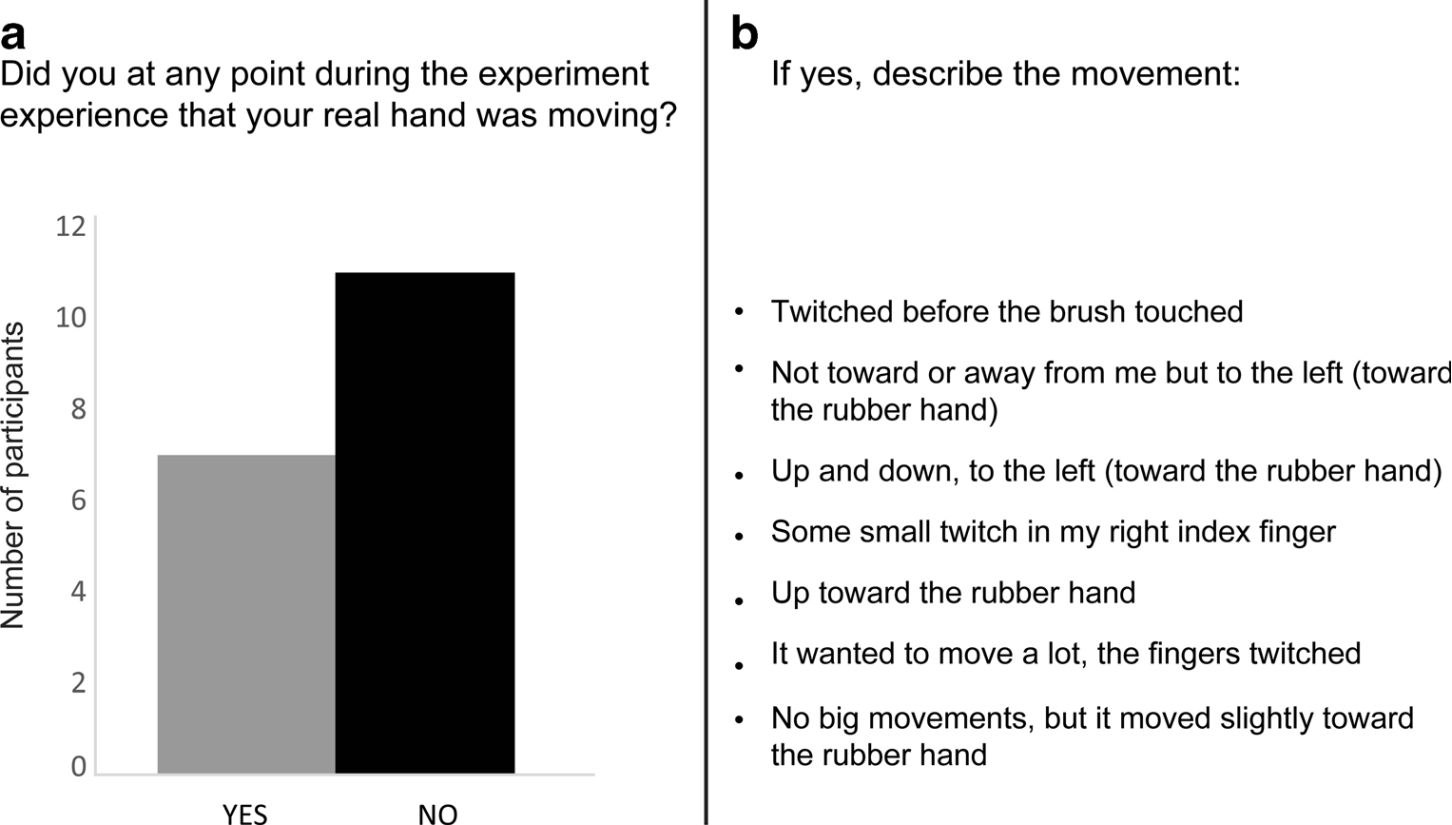
Postexperiment questionnaire results. (A) Responses to the forced choice question Q3 (see Table 2). (B) Participants’ responses to Q4. The responses to Q4 are translated from the original Swedish into English

The results of the questions administered after the main experiment showed that 11 participants experienced no movement (Fig. 9A). Seven of the participants reported having experienced some type of movement of their hidden right hand during the experiment. Critically, however, none of them described a movement that could be attributed to the mechanical displacement of the hand generated by the apparatus (i.e., away from the rubber hand in the horizontal plane). This is evident in Fig. 9B, in which we list the open-ended descriptions that the seven participants provided about their “movement sensations.” Thus, it is unlikely that the participants were aware of the movement as their hands were slowly displaced during the experiments.

## Discussion

We investigated the possible causal relationship between hand position sense and the rubber hand illusion. The main finding was that very slow mechanical displacement of the participant’s real hand during the induction of the rubber hand illusion, so slow that the participants were not aware of it, did not significantly alter the illusory experience of limb ownership. Specifically, we found that the perceived hand position (reflected in the proprioceptive drift) could be strongly affected by the external manipulation of hand position—toward or away from the model hand—without affecting the feeling of ownership or referral of touch measured by the subjective ratings on the questionnaires. Thus, these observations suggest that manipulating the sense of hand position during the rubber hand illusion does not influence the subjective illusion, which implies that proprioceptive drift either happens as a consequence of the illusion or constitutes an independent, parallel process. This conclusion has bearing on models of body ownership and central body representation.

The main novelty of the present study is that we examined the causal necessity of proprioceptive drift with regard to inducing a limb ownership illusion. Thus far, all available evidence has demonstrated that synchronous visuotactile stimulation in the rubber hand illusion is intimately linked to proprioceptive drift; that is, if you deliver the former, you are likely to observe the latter (Botvinick & Cohen, 1998; Costantini & Haggard, 2007; Guterstam et al., 2013; Kalckert & Ehrsson, 2012, 2014; Petkova & Ehrsson, 2009; Rohde et al., 2011; Samad et al., 2015; Tsakiris & Haggard, 2005). This has been used as an argument to defend the use of proprioceptive drift as a behavioral proxy for measuring the strength of the illusion. However, because it has been shown that it is possible to detect proprioceptive drift without the feeling of ownership, it has been proposed that although they have been reported to correlate, the two might constitute fundamentally different processes that are causally independent (Holle et al., 2011; Rohde et al., 2011). The present findings show that changing the sensed hand position either toward or away from the rubber hand had no influence on the strength of the rubber hand illusion. This suggests that proprioceptive drift does not play a fundamental causal role in producing the illusion, supporting the view that the subjective illusion and proprioceptive drift constitute two different processes.

In a previous study by Rohde et al. (2011), the authors argued that their results refute a causal link between proprioceptive drift and ownership, in the sense that the rubber hand illusion is not necessary for inducing the drift. Nevertheless, Rohde et al. were open to the possibility that the causal link might be in the reverse direction—that is, that proprioceptive drift might be a prerequisite for the illusion. However, our results suggest that proprioceptive drift is not a prerequisite for the illusion, and that relatively large mechanical manipulations of hand position sense do not affect the subjective illusion. Even so, we must note that our results merely demonstrate that it is possible to induce an illusion without it being accompanied by proprioceptive drift. It is not possible, on the basis of our data alone, to make any claims about whether proprioceptive drift is a completely separate process that correlates with the feeling of ownership or whether the ownership itself produces the drift. Considering that changes in proprioceptive drift have been observed in various conditions that do not involve changes in ownership, one could argue against the claim that ownership causes proprioceptive drift (Rohde et al., 2011). However, it remains possible that proprioceptive drifts in different contexts could result from different causal factors, and that the subjective rubber hand illusion is only one of these factors. In other words, the fact that hand position sense can drift in varying situations does not mean that the subjective rubber hand illusion cannot cause proprioceptive drift. To summarize, by interpreting our present findings in conjunction with the results that Rohde et al. (2011) obtained, we are inclined to conclude that proprioceptive drift is a process distinct from the core processes that underpin the subjective feeling of limb ownership in the rubber hand illusion.

To validate the rubber hand illusion setup employed in our experiments, we reproduced the classical proprioceptive drift toward the rubber hand in the static-hand conditions—that is, when the participant’s real hand remained immobile. Moreover, when comparing the synchronous and asynchronous static conditions, we reproduced the observation of a correlation between the subjective illusion and proprioceptive shift (Guterstam et al., 2013). The finding of significant proprioceptive drift in the classical static condition does not challenge the main conclusion of the present study; on the contrary, it serves as an important control that indicates that we successfully induced the rubber hand illusion in the static-hand conditions. This finding is in line with previous studies that have described both positive questionnaire responses and significant proprioceptive drift (or shift) toward the model hand in conditions in which the hand was not moved. The idea that is being challenged here is that recalibrating limb position toward the rubber hand is necessary for the feeling of ownership, and our results from the displaced-hand conditions refute this hypothesis. The small differences observed between the synchronous and asynchronous static conditions might have arisen as a consequence of ownership in the synchronous condition, or they might represent the disruption of the proprioceptive recalibration by asynchronous visuotactile stimulation proposed by Rohde et al. Interestingly, in the experimental condition in which the hand was mechanically displaced, we observed no difference in proprioceptive drift between the synchronous and asynchronous conditions and did not find any significant correlation between the subjective illusion and proprioceptive shift. One might wonder why a small synchrony-specific proprioceptive drift was not noted in the displaced-hand conditions and added to the mechanically induced changes in proprioception. We do not have a definite answer, but one possibility is that during the displaced-hand condition, the brain received more afferent proprioceptive inputs from muscles, joints, and stretching skin than in the static-hand condition. This extra inflow of afferent information would help to maintain a more accurate central representation of hand position and reduce the influence of vision on the final estimate. In the static condition—that is, when the hand was passive and occluded from view—illusion-induced proprioceptive drift toward the rubber hand could occur more easily because of the lack of dynamic proprioceptive feedback, meaning greater uncertainly of hand position, thereby increasing the weighting of visual cues for estimating hand position (van Beers, Sittig, & Gon, 1999; van Beers, Wolpert, & Haggard, 2002).

One concern that we had was that the participants might become consciously aware of the mechanical displacement of their real hand during the experiments. Given that proprioceptive drift in the classical rubber hand illusion occurs without any conscious sensation of limb movement (Botvinick & Cohen, 1998), it was important that the participants not perceive the mechanical displacement of their hidden right hand in the present study. To ensure that this was the case, the hand was moved sufficiently slowly (less than 0.3 deg/s) to ensure that the participants would not be able to detect the movement (Pickett & Konczak, 2009). As was evident from the denial of statement S4, which referred to movement sensations (see Figs. 2 and 5), the participants did not experience any movement of their hand during the experiment. Furthermore, we asked the participants to complete a postexperiment questionnaire (only in Exp. 2) to rule out putative movement sensations (Fig. 9). The postexperiment questionnaire results clearly show that the majority of participants (11 out of 18) did not experience any movement of their right hand. The remaining seven participants claimed to have experienced movement, but when they were asked to describe it, they did not describe a movement that could have been attributed to our external manipulation of their limbs (Fig. 9). Thus, their responses most likely represented confabulation, task compliance, or imagination.

Because the participants were unaware of the very slow movement of their hand in the displaced hand conditions, we argue that we were successful in our aim to simulate the proprioceptive drift that occurs in the rubber hand illusion with an immobile limb. However, principal differences remain between the purely “illusory” drift seen in the classical rubber hand illusion and the mechanical displacement of the participant’s hands. In the classical rubber hand illusion, the proprioceptive drift occurs without any manipulation of the actual limb position, whereas the mechanical displacement of the hand likely produced increased proprioceptive inflow to the brain from the muscle spindles in the arm as a consequence of the movement. From a theoretical point of view, this might be considered a problem when analyzing the mechanism of proprioceptive drift, because the two processes causing the changes in limb localization were different. However, proprioceptive drift is merely a change in hand position sense and so is the end result of the slow mechanical displacement. This change in proprioception toward the model hand is precisely what has been argued to be a necessary component for the occurrence of the rubber hand illusion. The mechanical displacement in our experiment thus mimicked proprioceptive drift for the purposes of our study: a change in the indicated hand position toward the rubber hand without any accompanying conscious sensations of limb movement.

How do the present results fit with existing models of body ownership? The results are difficult to reconcile with the original model proposed by Botvinick and Cohen (1998), in which the illusion is conceptualized as a three-way interaction between vision, touch, and proprioception. According to such a three-way connectionist model, a change in proprioception should influence the other factors in the model, and hence the illusion. Similarly, our findings cannot be explained by the more complex neurocognitive model of Tsakiris (2010). This model describes several so-called “critical comparisons” that take place during the processing of sensory and mnemonic information during the rubber hand illusion. The first comparison is between the visual form of the object (i.e., the rubber hand) and the preexisting reference model of the body. The second comparison is between the current state of the body (i.e., its posture) and the postural and anatomical features of the body part that is to be experienced as one’s own. Finally, the third comparison is between the sensory inputs—that is, between the seen and felt touches of the brush. Increasing or decreasing the correspondence in the second comparison, as was done in the present experiment when we increased or decreased the spatial distance between the rubber hand and the real hand, should presumably affect this key process, and hence the illusion; however, we did not observe such an effect.

The model of body ownership from Makin et al. (2008) places great emphasis on visuotactile integration in peripersonal space. The core idea is that the referral of touch to the rubber hand is mediated by visuotactile integration in the perihand space. This triggers the rubber hand illusion, which in turn triggers the shifts in peripersonal space toward the rubber hand, and thus gives rise to proprioceptive drift (Makin et al., 2008). Proprioceptive drift is conceptualized as arising because the synchronous visuotactile stimulation provides positive feedback that further adds to the weighing of vision over position sense in determining limb location (Makin et al., 2008). In the present experiment, the rubber hand was always presented within peripersonal space and never farther than 24 cm from the hidden real hand, thereby not violating the perihand space constraint of the illusion (Brozzoli, Gentile & Ehrsson, et al. 2012; Guterstam et al., 2013; Kalckert & Ehrsson, 2014; Lloyd, 2007; Preston, 2013) and making the present results fully explainable within this model (Makin et al., 2008). Rohde et al. (2011) modified the model by Makin and colleagues by stating that the asynchronous visuotactile stimulation might provide negative feedback, which inhibits existing processes of visuo-proprioceptive integration. The results of our experiment do not contradict this model, but we also did not attempt to replicate the “vision-only” condition, in which participants view the rubber hand with no visuotactile stimulation (Rohde et al., 2011). Hence, we cannot determine whether the asynchronous condition eliminates proprioceptive drift or the synchronous condition triggers it (or a combination of both).

Finally, the present results appear to be consistent with the Bayesian causal inference model of the rubber hand illusion that was recently proposed by Samad et al. (2015). In this model, inference of a common cause is made from visual, tactile, and proprioceptive signals (i.e., an owned rubber hand sensing the touches of the paintbrush; Samad et al., 2015). According to the predictions of this model, the illusion begins to vanish once the distance between the rubber hand and the participant’s hand approaches 30 cm, which was never the case in our present experiments. Furthermore, the model indicates that the inference of a common cause of the visual, tactile, and proprioceptive signals (i.e., the ownership illusion) will lead to greater proprioceptive drift in the synchronous than in the asynchronous static-hand conditions, which is in line with our results.

What do the present results state with regard to the empirical question of how best to objectively quantify the rubber hand illusion? It should be clear to the reader that one should not rely on the proprioceptive drift measure alone. A single direct measure of the rubber hand illusion has not been agreed upon in the literature, and therefore most researchers use a combination of measures, typically one subjective measure (often in the form of visual analogue rating scales on questionnaires) combined with one or more objective measures (de Vignemont, 2011; Ehrsson, 2012). Proprioceptive drift (or shift) is a widely used objective behavioral measure, but others include threat-evoked skin conductance responses (Armel & Ramachandran, 2003; Guterstam et al., 2013; Guterstam et al., 2011; Petkova & Ehrsson, 2009), local temperature changes of the hand (Hohwy & Paton, 2010; Moseley et al., 2008; but see Rohde et al., 2013, for a critical view), and changes in reactions times in the cross-modal congruency task (Pavani, Spence, & Driver, 2000; Zopf, Savage, & Williams, 2010). We argue that proprioceptive drift can be used as a valuable *indirect* measure of the rubber hand illusion if it is used with caution in well-controlled rubber hand illusion experiments when the real hand is immobile (or when only one finger is moved; see Kalckert & Ehrsson, 2012). The proprioceptive drift measure has some advantages compared with other objective measures, in that it is easy to administer and often correlates with the subjective illusion (as the proprioceptive shift did in our static conditions; see Fig. 8). The threat-evoked skin conductance response suffers from the facts that not all participants display changes in skin conductance response (null responders) and that the response attenuates after repeated threats, even in good responders. The changes in hand temperature reported by Moseley and colleagues appear to be quite difficult to reproduce (Paton, Hohwy, & Enticott, 2011; Rohde et al., 2013; van Stralen et al., 2014), and temperature’s functional relationship with the subjective illusion is not yet fully understood. Finally, the presentation of visual and tactile target stimuli near the rubber hand for speeded reactions in the cross-modal congruency task can interfere with the rubber hand illusion itself, because it introduces visual and/or tactile stimuli in proximity to the rubber hand, thus making this manipulation difficult to use in many paradigms. Overall, we argue that the proprioceptive drift measure can be a valuable tool, when it is used in well-established paradigms together with other measures and the results are interpreted with caution.

In summary, in this study we investigated the link between the feeling of ownership and proprioceptive drift in the rubber hand illusion. In particular, the causal role of proprioceptive drift in generating the rubber hand illusion was examined by employing an apparatus that was able to relocate the participant’s hidden limb without the participant noticing this movement, while the illusion was being induced. The results show that the strength of the illusion as measured by subjective questionnaire ratings was not influenced by changes in sensed hand position toward or away from the rubber hand. These findings are not consistent with the hypothesis that proprioceptive drift is a necessary causal factor for producing the illusion. Rather, they suggest that proprioceptive drift is an independent process that, under certain conditions, is correlated with or caused by the subjective illusion. These results advance our understanding of the perceptual processes that underpin the rubber hand illusion.
